# A Case Report of Atraumatic Tooth Extraction Followed by Ridge Preservation for Implant-Supported Prosthetic Rehabilitation Using an Alloplastic Bone Graft

**DOI:** 10.7759/cureus.50776

**Published:** 2023-12-19

**Authors:** Shrishti S Salian, Khushboo K Durge, Prasad V Dhadse

**Affiliations:** 1 Periodontics and Implantology, Sharad Pawar Dental College and Hospital, Datta Meghe Institute of Higher Education and Research, Wardha, IND

**Keywords:** t-prf, alloplastic bone graft, implant, ridge preservation, atraumatic extraction

## Abstract

Exodontia is a painful treatment that frequently causes the alveolar bone and surrounding soft tissues to be immediately destroyed and lost. With regard to the amount of resorption taking place after extraction, various treatment protocols aimed at preventing or decreasing alveolar ridge collapse have been presented over the past three decades. Ridge preservation is a clinical technique used to prevent the socket walls' bone resorption after tooth extraction. A 43‑year‑old female patient with a non-significant medical history visited the Department of Periodontology and Implantology with a chief complaint of a decayed tooth in the upper left back region (26) for three years and wants to get it replaced. The treatment option that was given to the patient was atraumatic extraction, followed by ridge preservation. A cautious and conservative treatment strategy is necessary to preserve the oral structures as they currently exist and are intact for a successful outcome; careful case selection and thorough treatment planning are crucial. Atraumatic tooth extraction is a procedure used to delicately remove a tooth while upholding the fundamental principles of preserving the surrounding bone and gingival structure. This will ultimately maximize the success of implant placement in terms of appearance and functionality.

## Introduction

Exodontia is a painful treatment that frequently causes the alveolar bone and surrounding soft tissues to be immediately destroyed. Following that, a complicated series of biochemical and histologic occurrences take place during the healing process, which further causes physiologic changes in the architecture of the alveolar bone and soft tissues [1]. Over the past three decades, a number of therapeutic approaches targeted at preventing or minimizing alveolar ridge collapse have been offered, taking into account the resorption occurring following exodontia. A therapeutic procedure called "ridge preservation" is performed to stop the resorption of bone from the socket walls following tooth extraction. As the saying goes, "We should replace what we take out with what we put back in" [2].

Influence of alveolar ridge anatomy on esthetics

After uneventful extraction socket healing, a normal ridge is one that still resembles the alveolar process in general. A ridge that is partially or entirely devoid of teeth may nonetheless preserve the general size and density of bone that was present in the dental arch before the teeth were lost [3]. The ridge shape can be defined as the geometric form of an alveolar process or the residual ridge [4].

The smile zone comprises the lower third of the face and has been defined by its superior limit which is bordered by a second horizontal line drawn along the base of the nose. Smile zone can be further divided into superior, middle and inferior thirds while in response. The superior third of the smile zone comprises the alae of the nose, the nasolabial sulcus and the philtrum. The middle third of the smile zone extends from the mucocutaneous border of the lower lip to one-half the distance between it and the base of the chin. The lower third of the smile zone comprises the mandibular symphysis and associated musculature forming the chin [4].

## Case presentation

A 43-year-old female patient with an insignificant medical history visited the Department of Periodontology and Implantology with a chief complaint of a decayed tooth in the upper left back region (26) for three years and wants to get it replaced. On clinical examination, the preoperative intraoral periapical (IOPA) revealed root stumps at 26 regions. The treatment option that was given to the patient was atraumatic extraction, followed by ridge preservation with demineralized freeze-dried bone allograft (DFDBA) and titanium platelet-rich fibrin (TPRF), followed by implant placement after 12-14 weeks.

**Figure 1:**
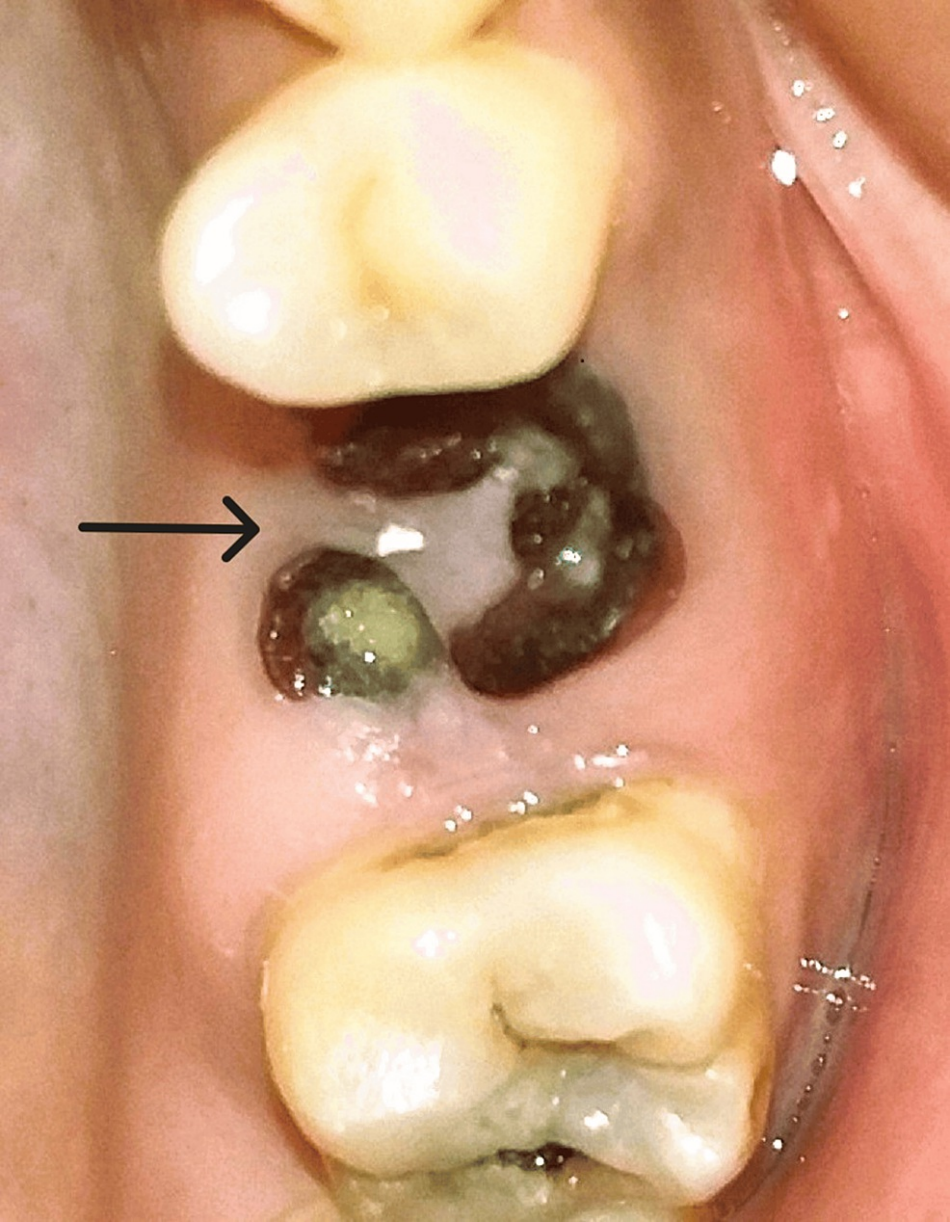
The pre-op clinical view

**Figure 2:**
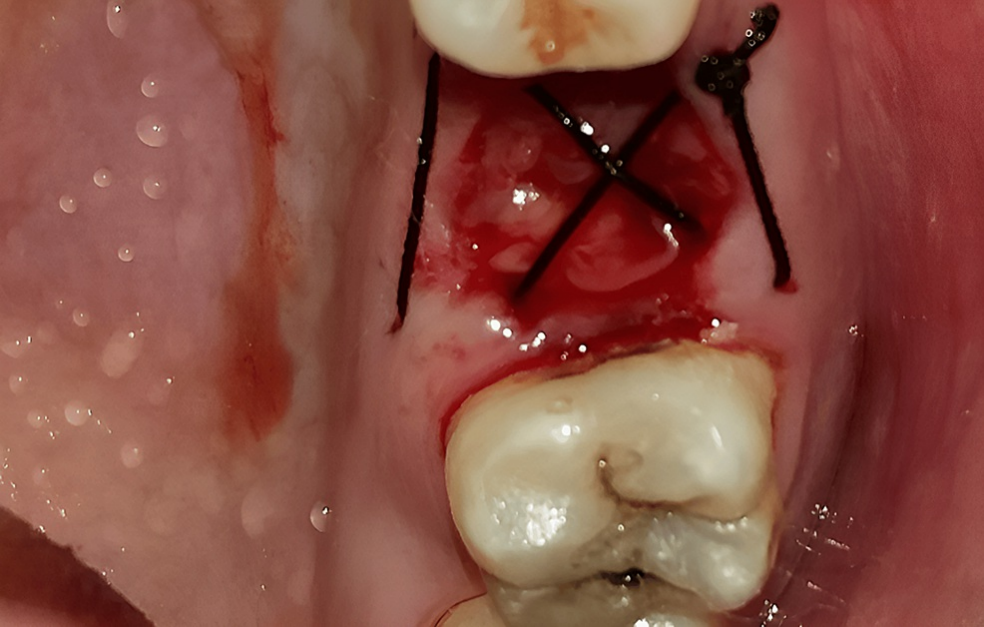
The site was left exposed with the purpose of allowing it to recover later.

**Figure 3:**
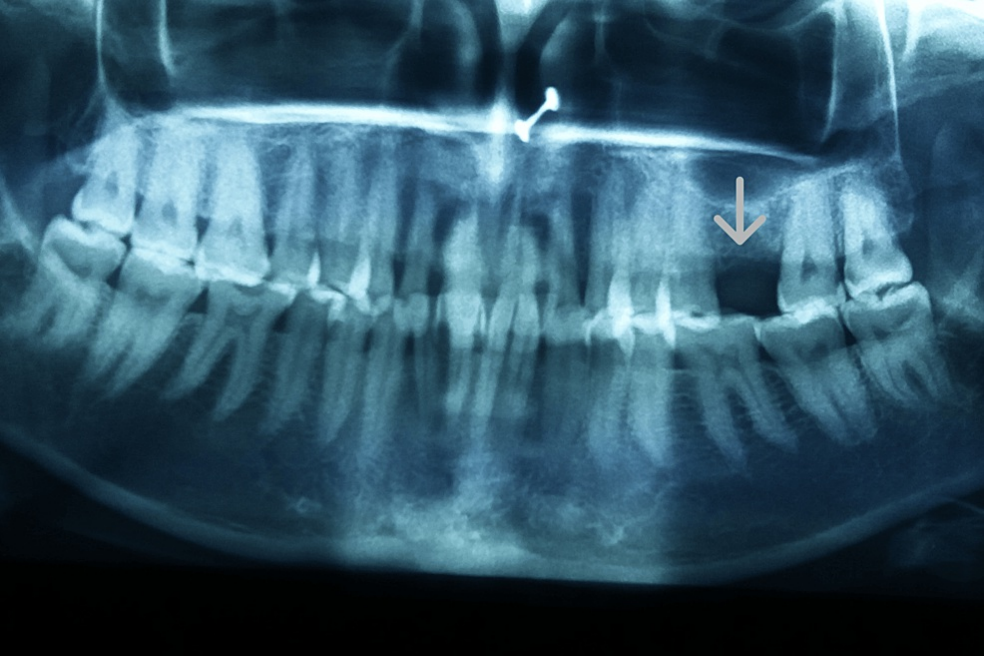
OPG revealed that the graft material had revealed densification. OPG: orthopantomogram

The procedure was performed under local anaesthesia. Figure 1 shows the preop clinical view. A delayed type of implant placement was planned for treatment, by excising the decayed tooth while simultaneously grafting the socket and placing the implant after a healing period of 12 weeks. It was done after a thorough clinical and radiographic assessment. Under local anaesthesia, tooth extraction was carried out without elevating the flap. Using a number 15 blade, a crevicular incision was performed, and periodontal ligament (PDL) fibres were cut on the mesial and distal sides. Elevators were used to gently luxate the teeth. The surrounding tissues were not injured. Following exodontia, complete granulation tissue debridement of the socket was done using bone curettes. A fully resorbable alloplastic bone substitute, DFDBA was placed. After the graft was applied, care had to be taken not to overfill the socket because doing so could then lead to the exposed coronal particles being sequestrated or dispersion of the entire graft mass. TPRF was used to accelerate the in-situ healing and further compact the graft particles. To provide soft tissue stability, a cross-mattress tension-free 3.0 non-absorbable silk suture was applied over the location after it had been covered with a hemostatic dressing material. Without establishing primary closure, the site was left exposed with the purpose of allowing it to recover later (Figure 2). It was recommended to take antibiotics and use a mouthwash with 0.2% chlorhexidine for seven days. One week after surgery, the suture removal was done. Postoperatively, the healing was uneventful, and the bone transplant particles were not lost as the area was gradually covered by newly developed soft tissue. An orthopantomogram (OPG) at this time revealed that the graft material had revealed densification after 12 weeks, leading to bone regrowth at the location (Figure 3) [5].

**Figure 4:**
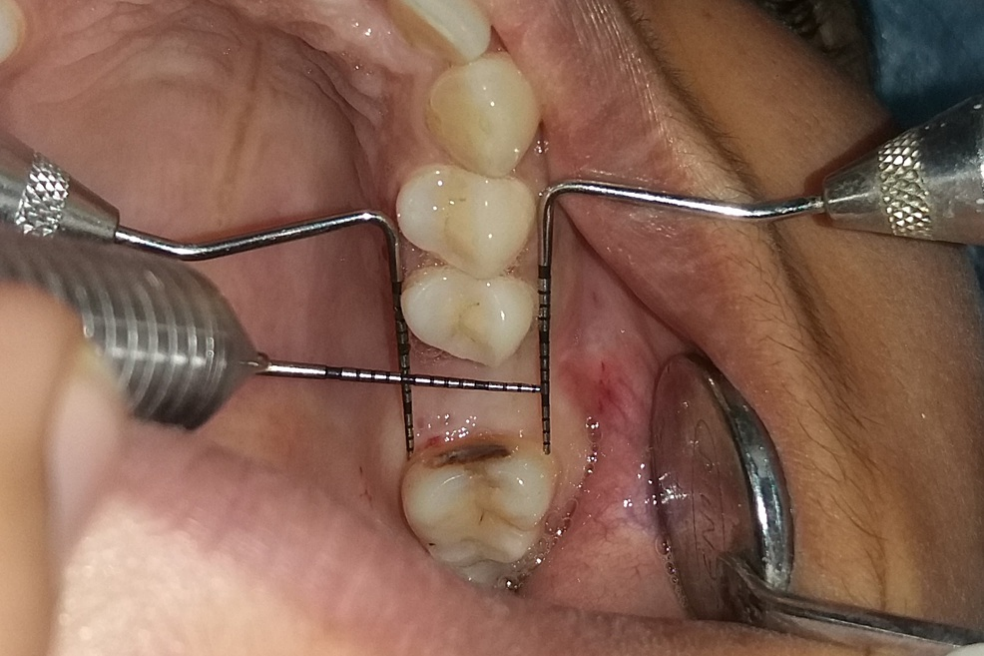
Measurements taken before implant placement.

**Figure 5:**
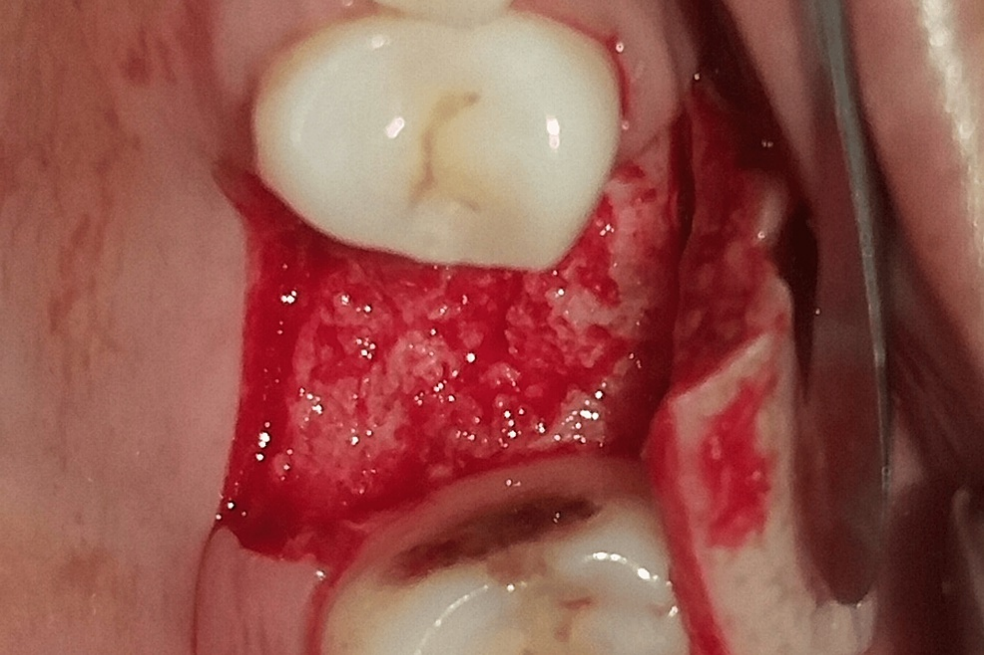
Full-thickness flap was elevated.

**Figure 6:**
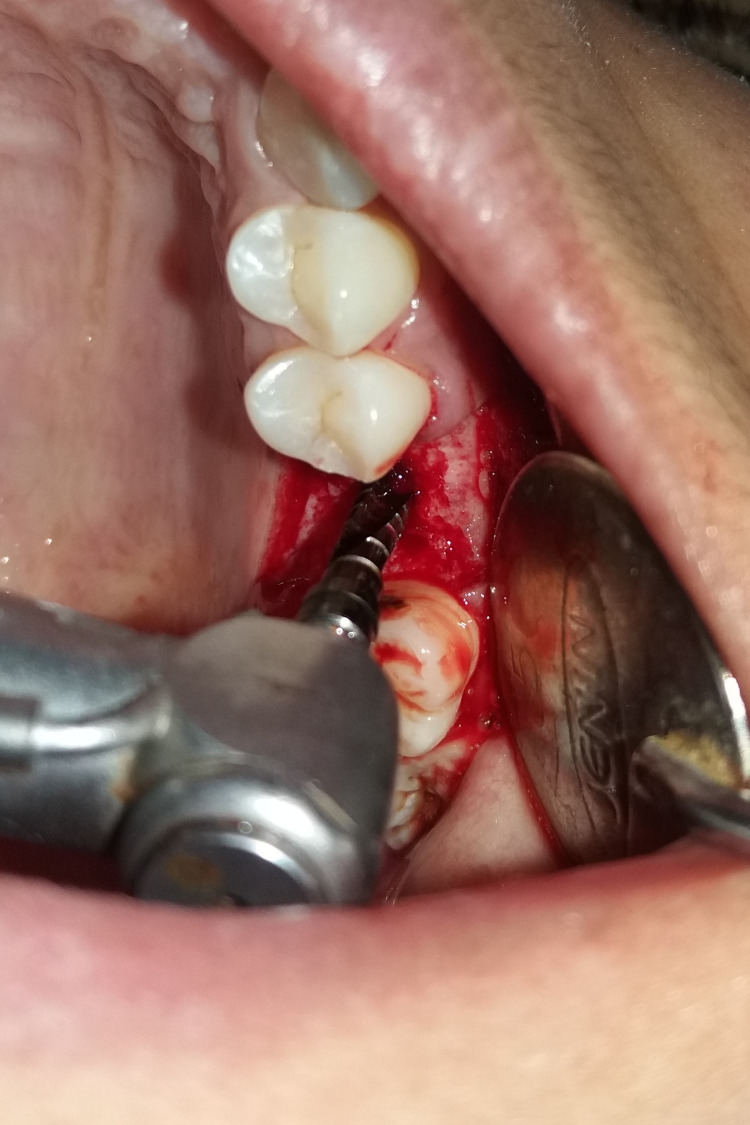
An implant of size 3.5x10 mm was placed.

**Figure 7:**
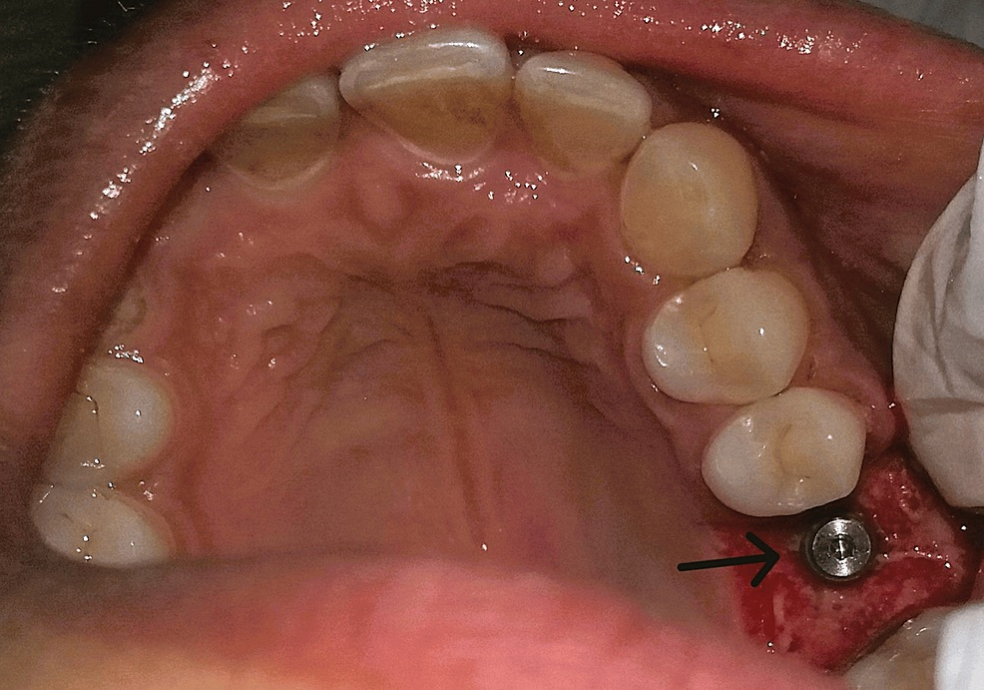
Placement of cover screw

**Figure 8:**
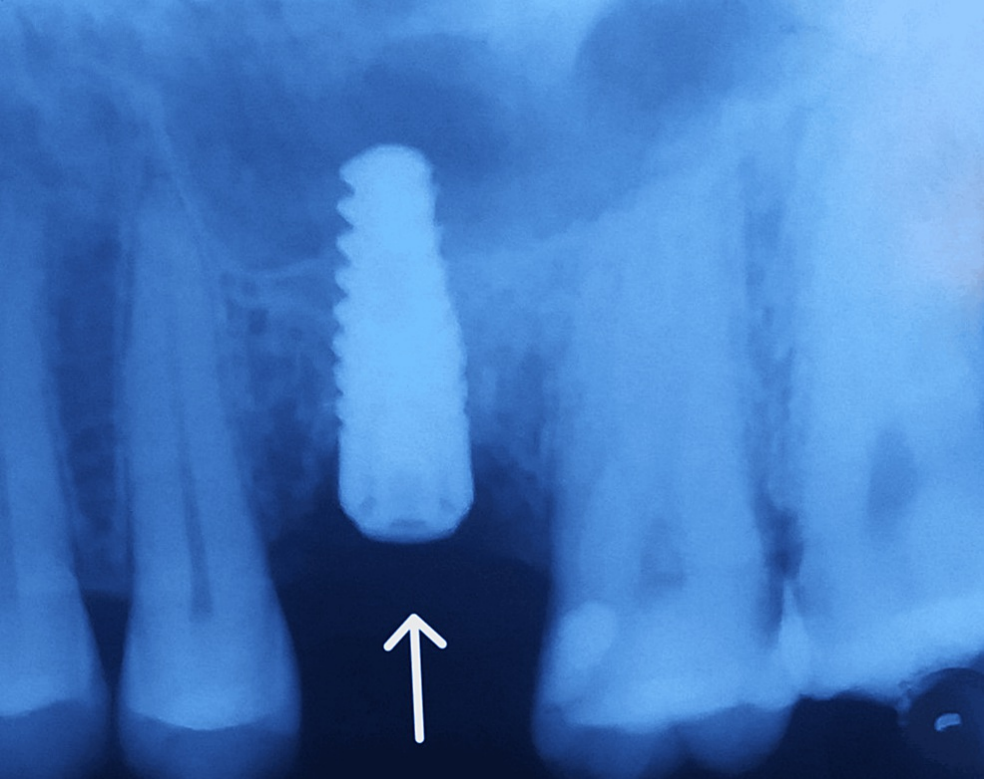
Post-operative IOPA suggesting that the implant placement is correct. IOPA: intraoral periapical

**Figure 9:**
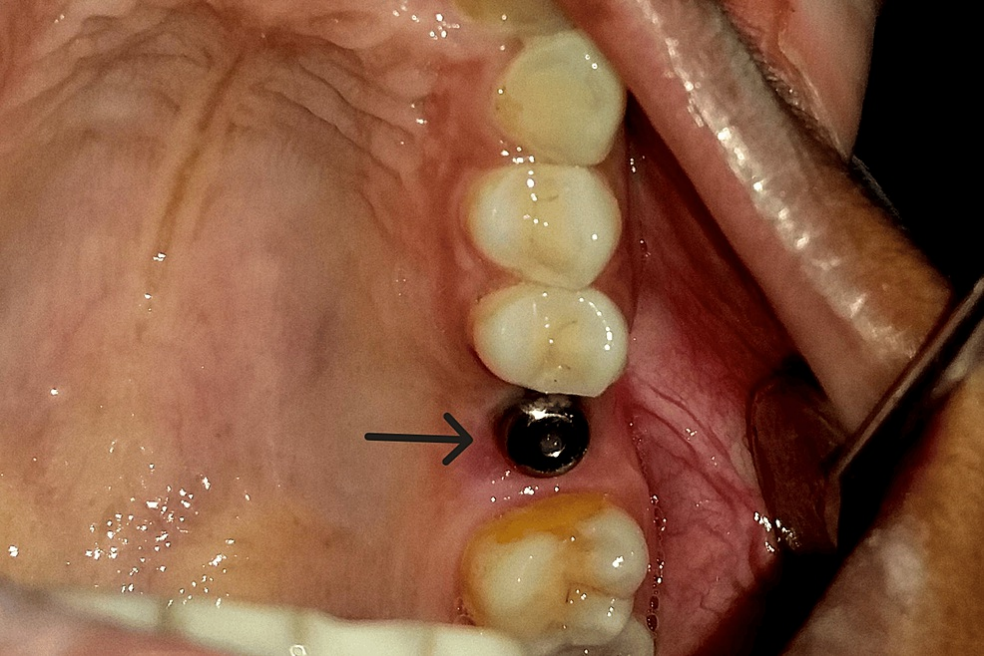
Second-stage implant surgery

A mid-crestal incision in 26 regions was given, following which a full-thickness flap was elevated (Figure 4 and Figure 5). The implant of size 3.5x10 mm was placed (Figure 6) followed by the placement of the cover screw (Figure 7). Suturing was done using a 3.0 silk suture for the approximation of tissues. An immediate post-operative IOPA suggested that the implant placement was correct (Figure 8). Just as in the first phase, the patient was prescribed antibiotics and analgesics for five days. There were no post-operative complications and the suture removal was done after two weeks. The second phase of the implant surgery was done following 12 weeks of osteointegration (Figure 9) and the patient was referred to the Department of Prosthodontics.

**Figure 10:**
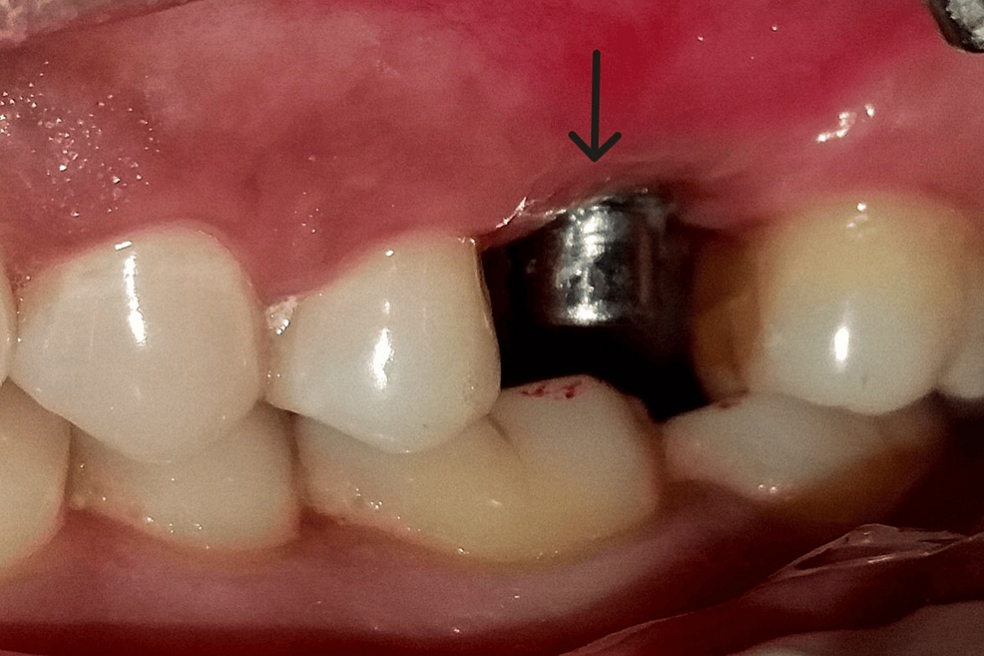
Healing abutment placed.

**Figure 11:**
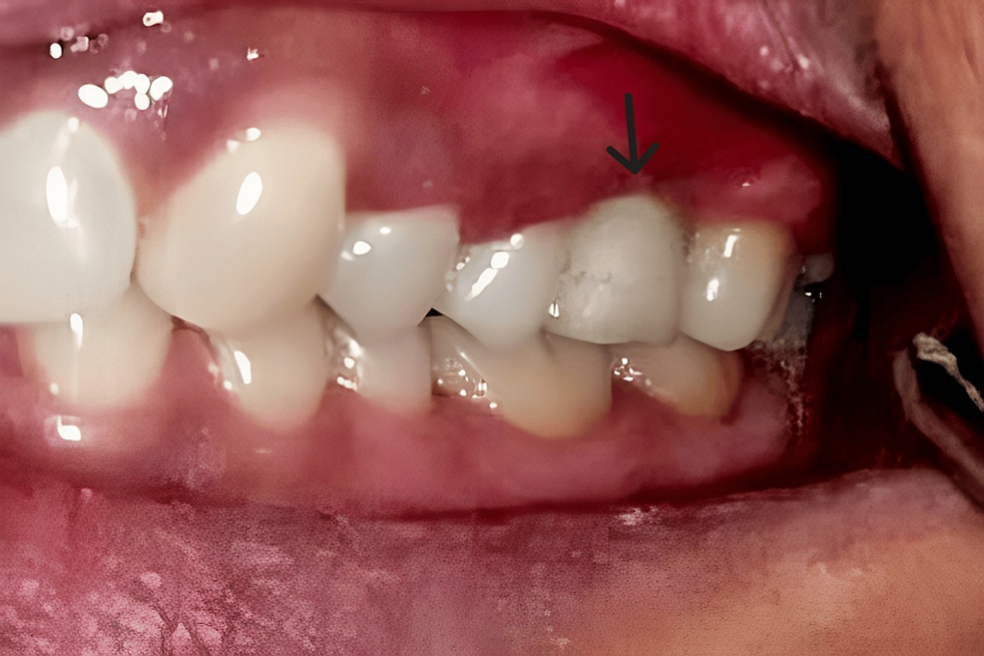
Definitive crown was cemented.

**Figure 12:**
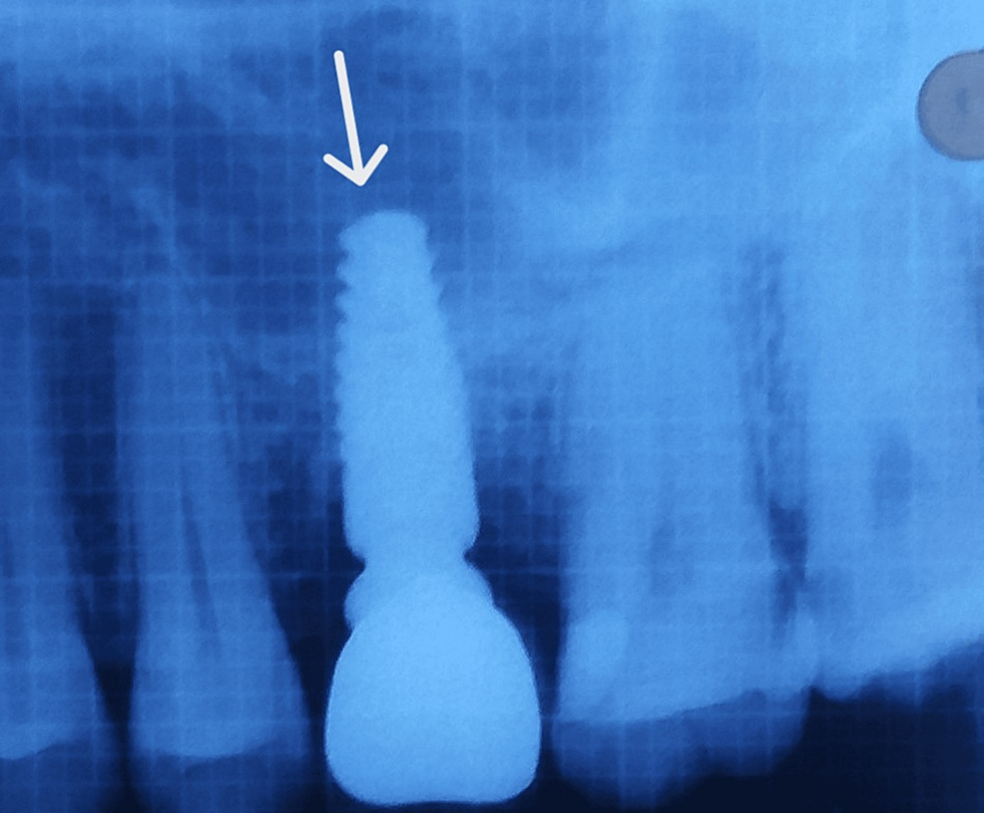
Immediate post-op IOPA IOPA: intraoral periapical

**Figure 13:**
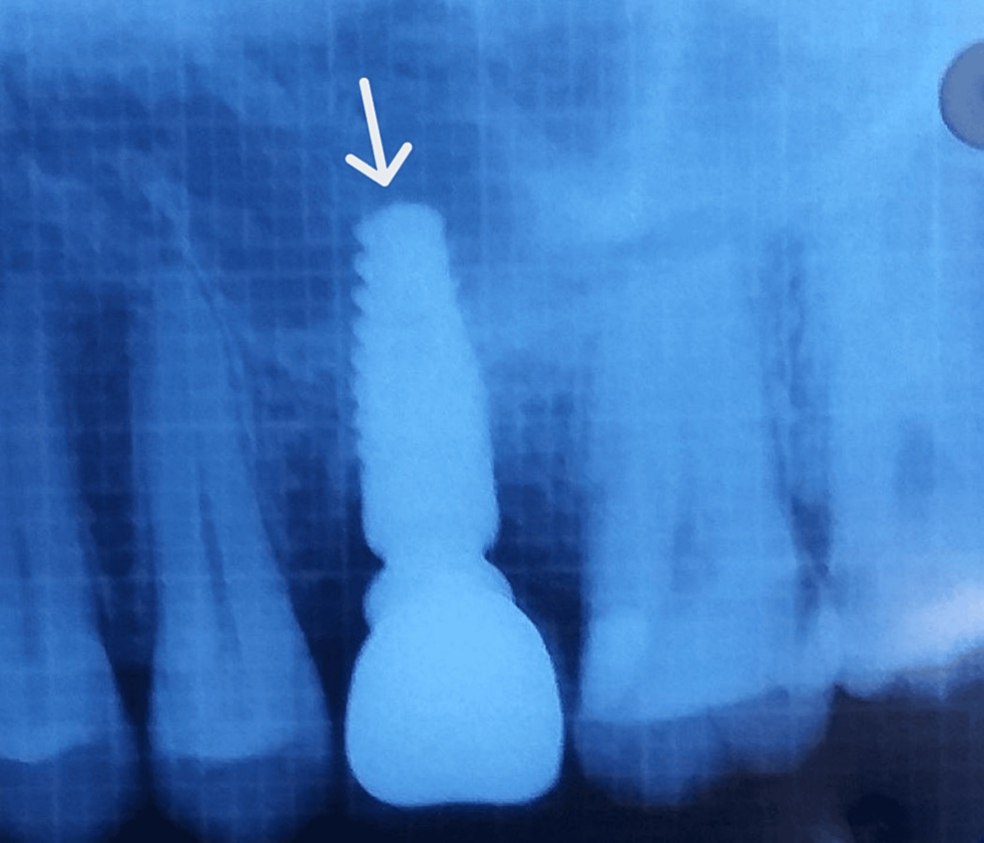
Post-op IOPA taken nine months later. IOPA: intraoral periapical

To create an implant-level impression, the healing abutment was taken out and the impression coping fitted into the implant (Figure 10). To create the functioning cast, the closed tray impression technique was used to create an impression using a polyvinyl siloxane material, which was then poured once the implant analogue was secured. An interocclusal record, an impression of the opposing teeth, and shade selection were completed. The anterior definitive crown was cemented over the definitive implant abutment at the most recent consultation (Figure 11). An immediate post-op IOPA was suggested that the prosthesis placement was correct (Figure 12) and a post-op IOPA was taken nine months later which showed no complications with the implant or the prosthesis (Figure 13).

## Discussion

A minimally intrusive procedure was used in this instance. Without raising a flap, extraction and socket grafting were carried out, and neither a flap nor a barrier membrane was incorporated to cover the augmented site. This technique was selected in order to facilitate the natural growth of fresh soft tissue over the post-extraction site and to try not to disturb the mucogingival interface [5]. Diverse variations have been suggested for the ridge preservation protocol like the socket plug technique. Although the socket plug method introduced a ridge preservation treatment without the need for flap closure primarily, the postoperative discomfort brought on by the graft did not significantly reduce. Shortly after, the Bio-Col procedure was introduced, which followed a similar concept as the socket shield operation but substituted organic bovine bone mineral for bone and used a collagen plug to block the socket in place of the soft tissue graft. Due to the absence of flap elevation or graft harvesting, this novel concept significantly decreased postoperative discomfort [6].

For filling major bony deficiencies brought on by cysts, tumours, alveolar resorption, and periodontal bony defects, autogenous bone has been referred to be the gold standard, as they leave insufficient bone for implant placement. There are now two types of allograft bone available: demineralized bone matrix (DBM) and mineralized bone tissue. DBM is processed cadaveric bone that has had the mineral component removed, leaving a scaffold of collagen and other growth-factor proteins that have been demonstrated to promote the production of new bone. To maximize the osteoinductive characteristics of bone allograft, demineralization is required. When appropriately processed from a suitable host, DBM offers an osteoconductive matrix and has the potential to be osteoinductive [7]. Majzoub et al. (2019) examined the impact of several bone substitutes used to preserve the alveolar ridge on the changes in post-extraction dimensions. The authors concluded that using a bone grafting material to preserve the alveolar ridge slows down the degradation process after exodontia [8].

Periodontal surgery has long made use of the idea of establishing a barrier to stop epithelial cells from migrating into the wound and give time for bone growth. An exclusionary barrier membrane promotes selective osteogenic cells to proliferate since it prevents the downgrowth of soft tissue. TPRF is a third-generation platelet concentrate that helps in the regeneration of tissue. Tunali et al. created TPRF, which prevents any adverse effects caused by dry glass or tubes with glass coating [9]. The use of titanium tubes in the preparation of TPRF demonstrated more polymerized fibrin formation with a longer resorption rate in the tissues, demonstrating that TPRF provides firmer fibrin. This is because titanium appears to be more effective at activating platelets than silica activators in glass tubes [10,11].

In 1976, Schulte and Heimke reported immediate implants for the first time in a clinical study. The purpose of the "immediate implant" is to stop bone resorption after exodontia. This technique reduces the healing time by preserving the ridge's height and dimension. "Immediate implant placement" in the aesthetic zone yields positive results. It was noted in systematic reviews that, to reduce risks, "immediate implant" insertion should ideally be carried out in a meticulously chosen group of patients. Orofacial soft tissue flattening and facial mucosal recession could be drawbacks for immediate insertion in less favourable instances [12].

A large percentage of oral fluid exposure and an infected extraction socket also exist, which raises the possibility of bacterial infection and, consequently, compromised bone development. It is best to postpone implant surgery if periapical infection is present. Siciliano et al. in their study stated that inadequate soft tissue approximation is also analogous with "immediate implant placement" [13]. The majority of clinicians now refrain from placing endosseous dental implants at diseased sites right away as a result of this clinical experience. Delay in implant placement is therefore encouraged since it results in improved flap management. Compared to implants that are placed immediately, they show a decreased incidence of soft tissue dehiscence in the "membrane-based regeneration". A completely closed flap and an undisturbed clot are prerequisites for optimal bone repair. The method raises the soft tissue level and permits the resolution of acute infections [14].

According to the available data, implant placements that are delayed may have a marginally greater survival rate than those that are done immediately, but there is also a chance that the former will be less aesthetically pleasing, require longer treatments, and cause more discomfort for the patient [15].

## Conclusions

Atraumatic tooth extraction is a procedure used to delicately remove a tooth while upholding the fundamental principles of preserving the surrounding bone and gingival structure. A cautious and conservative treatment strategy is necessary to preserve the oral structures as they currently exist and are intact for a successful outcome; careful case selection and thorough treatment planning are crucial. An essential component of our practice is selecting the most effective treatment strategy for each patient. The physician will be able to provide the best care by knowing the many alternatives for ridge preservation and their benefits and drawbacks. This will ultimately maximize the success of implant placement in terms of appearance and functionality.
